# Mobile-Based Ecological Momentary Intervention for Improving Physical Activity in Adults Without Regular Physical Activity: Pilot Randomized Controlled Trial

**DOI:** 10.2196/79360

**Published:** 2025-12-04

**Authors:** Takeyuki Oba, Chihiro Moriishi, Keisuke Takano, Kentaro Katahira, Kenta Kimura

**Affiliations:** 1 Human Informatics and Interaction Research Institute, National Institute of Advanced Industrial Science and Technology, 1-1-1 Higashi, Tsukuba, Ibaraki, 305-8566, Japan, 81 80-2202-0737; 2 Faculty of Letters, Hosei University, Chiyoda-ku, Japan

**Keywords:** ecological momentary intervention, ecological momentary assessment, physical activity, motivation, heart rate reserve

## Abstract

**Background:**

The ecological momentary intervention (EMI) is one of the most promising digital—primarily mobile—interventions to enhance physical activity (PA) and other health behaviors. It is a combination of ecological momentary assessment (EMA), in which participants are prompted to indicate their momentary states and ongoing behaviors in daily life, and the just-in-time delivery of interventions tailored to the EMA responses. The EMI has typically been implemented in message-based interventions (eg, activity recommendations tailored to users’ physical locations), but its efficacy and feasibility have not been sufficiently established because of the variability in design and implementation.

**Objective:**

This pilot, two-arm, parallel-group randomized controlled trial aimed to be an exemplar of EMI for improving PA and establishing efficacy and feasibility among adults without a habit of PA.

**Methods:**

A total of 40 participants (23 women; mean age 45.40, SD 10.50 years) were recruited from among community dwellers in northeast Japan and randomly allocated to the EMI or control group. Each participant wore an activity tracker to monitor their daily step count and heart rate (HR) for 4 weeks (fully automated). Simultaneously, they responded to EMA questions about the current weather, location, and social context 3 times during the daytime and an additional evening question about motivations for PA each day. Only the EMI group received messages tailored to their responses to EMA, recommending more active alternative behaviors suited to EMA-reported contexts.

**Results:**

Participants wore a Fitbit device for 90.3% (21.66/24 hours per day) of the study period (mean 90.3, SD 10.0), and no dropouts were observed. The EMI group showed no significant improvement in the self-reported amount of PA (*P*=.44), step count (*P*=.24), or motivation for PA (from *P*=.11 to *P*=.91) compared with the control group. However, the EMI group showed a significantly larger increase in the minutes of 40% HR reserve, a measure of moderate or high intensity of PA (mean 16.03, 95% CI 3.76-28.29; Cohen *d*=0.20‐0.41; *P*=.02 for the follow-up weeks). The intervention was rated as marginally useful and satisfactory, and approximately half of the participants expressed a willingness to continue the intervention. The timing of the EMA prompting was considered inappropriate.

**Conclusions:**

These findings suggest that the EMI with messages tailored to EMA-reported contexts was not effective in increasing the amount or motivation for PA but may increase the intensity as assessed by the HR. The intervention aimed to help individuals implement small but slightly more active behaviors in their daily routine, which may not accompany prominent body movements but may be reflected in the increased HR. Marginal feasibility indicates that the intervention has sufficient scope for improvement, particularly in terms of prompt timing.

## Introduction

### Background

The rapid growth of mobile technologies has expanded the accessibility and applicability of health behavior change interventions that are delivered remotely, automatically, and ubiquitously to users in need. The ecological momentary intervention (EMI) is a mobile intervention that offers real-time support tailored to the dynamically changing states of users in daily life [1,2]. It often incorporates ecological momentary assessment (EMA) to determine the support offered at each moment, for which users make real-time or near–real-time self-reports via a mobile device about their current behavior, context, emotions, beliefs, and experiences [3]. An EMI system then selects the seemingly best intervention according to the EMA responses; for example, a system generates a message recommending outdoor activities to enhance physical activity (PA) when the weather is good and a user reports feeling energetic. A systematic review [1] found that coping strategy suggestions, including behavioral activation (eg, doing a short productive chore, such as loading the laundry or cleaning a room) [4] and restructuring negative thoughts [5], are the most typical interventions offered by EMIs, followed by motivational (eg, positive reinforcement and supportive messages) and information (eg, customized graphs) feedback [1]. For instance, an EMI trial delivered congratulatory and supportive messages when participants reported, in response to an EMA prompt, that they had successfully enacted a healthy behavior (eg, avoided sugar-sweetened beverages) [6]. Another trial [7] delivered 3 text messages each day on how to replace sitting time with a more active behavior (eg, standing up 5 times) with the sending schedules as well as the message contents and tones being adjusted to individual participants’ preferences. Participants provided real-time feedback about how they responded to each message, which was used to update the tailoring rule to generate messages. To date, various forms of implementations, designs, and system architectures have been explored and tested across trials, targeting different health issues, including dietary behavior, PA, and substance use [8-11]. Studies have provided preliminary support for EMI’s feasibility and efficacy; however, EMI remains an emerging area of research that calls for more empirical evidence [1].

The EMI has gained particular attention from researchers and practitioners working on PA. It is widely recognized that PA reduces the risk of serious health conditions [12], but a lot of people still fail to initiate and maintain PA. Providing health information may not suffice, and theories have articulated key aspects to trigger health behavior change. The capability, opportunity, motivation, and behavior (COM-B) model is one of the most pronounced frameworks, highlighting the following three determinants of PA: (1) physical and psychological capability (eg, having sufficient physical stamina to sustain PA and understanding why PA helps with health management), (2) opportunity (eg, having safe environments for PA and economic stability and receiving support from family and friends), and (3) motivation (eg, considering the long-term health benefits of PA and reducing emotional barriers like fear, stress, or fatigue) [13]. A health behavior change is triggered when these 3 are successfully implemented, and EMI is an ideal design to achieve it. This includes sending a prompt when a moment of opportunity arises, and prompting can be repeated as much as needed with almost no cost for human experts. Additionally, the adaptive nature of EMI concerns the appropriateness of the prompt, which is tailored to the moment or situation that the users are in (eg, not recommending outdoor activities during bad weather). Such tailoring would improve the acceptability of prompts and efficiently motivate individuals to engage in healthy behavior [14].

Regardless of its theoretical advantages, mixed evidence has been found for the efficacy of EMI for PA [15]. The EMI trials, typically designed to remind participants of their current (inactive) states and alternative active behaviors, have established positive effects on proximate outcomes, such as awareness [16], motivation, capability [17], and reduced computer use associated with sedentary behavior [18]; however, these effects did not transfer to PA; participants became more motivated, capable, and aware of opportunities, but their PA levels remained unchanged even after completing the intervention [19].

An important question arises here: How can EMI have an effect on a distal physical outcome? We kept two key aspects in mind when conceptualizing this study. First, regarding the intervention design, we followed Van Dantzig et al [20], who highlighted three principles for a successful EMI: (1) the messages should be scheduled to shoot actionable or reflective moments of the participants; (2) the message content and recommendations should be relevant and feasible given the current context and state of the user; and (3) the message format and delivery channels should fit the real-time, anytime-anywhere nature of the intervention (eg, concise and digestive texts and easy instructions resulting in an immediate reaction). These aspects were implemented in the EMI. Messages were sent 3 times a day (morning, afternoon, and evening) to increase the likelihood of accessing a moment of opportunity. Each message included activity recommendations tailored to the participants’ weather and location, which have been used as tailoring variables in former just-in-time adaptive intervention (JITAI) trials [21]. In addition, we incorporated the participants’ social contexts into the EMA to tailor the messages, encouraging participants to work together if someone else was present at the moment. Studies have shown that social components (eg, collaborating with or competing against peers) significantly contribute to behavior change interventions [22] but have rarely been implemented in EMI. We expected that the combination of these three tailoring variables (ie, location, weather, and social context) would allow for capturing individual participants’ contexts in higher resolution than former trials, which is, thereby, an opportunity to tailor each recommendation message to fit in individual contexts more closely. Also, the social addition would help participants become aware of the opportunities for using social resources to initiate and maintain their engagement with PA [21].

Second, we focused on the fact that the effect of EMI may not always be reflected in changes in the amount of PA or step count. The amount (often assessed by an accelerometer) is not the only determinant of PA intensity. For example, a study suggested that the percentage heart rate reserve (HRR), which is closely related to oxygen consumption and PA intensity [23,24], is significantly higher during occupational than leisure time PA. Furthermore, the percentage HRR shows a pronounced difference between sitting and standing, but no prominent difference between standing and moving or walking [25]. Existing EMIs typically suggest that participants implement small changes in daily behaviors requiring less effort (eg, walking faster and standing when a long sedentary time is detected) [15,26]. In other words, EMIs are not necessarily designed to increase the amount of PA or steps; however, such small behavioral changes may induce cardiovascular responses, interpreted as increased PA intensity. Therefore, we included heart rate (HR) as an exploratory outcome to test the efficacy of the EMI.

### Objectives

This study was a pilot, 2-arm, parallel-group, randomized controlled trial testing the efficacy and feasibility of message-based EMI for improving PA among adults without a PA habit. Our EMI was characterized by messages and activity recommendations tailored to participants’ responses to momentary contextual questions embedded in the EMA. In response to each EMA prompt, the participants reported the weather, location, and social interaction with others at that moment, and this information was used to immediately generate a message and recommendation delivered on a smartphone. We hypothesized that participants in the EMI group would show larger increases in the amount and intensity of PA as well as motivations for PA (as assessed by self-report questionnaires and wearable activity trackers) than those in the control group, who received real-time feedback on step count and HR but no tailored messages. In the exploratory analyses to clarify the appropriateness of the EMI recommendations, we tested (1) how likely participants were to accept the activity recommendations, (2) what activities were most likely to be accepted, and (3) whether the frequency of acceptance was associated with an increase in PA and motivation.

## Methods

### Participants

The participants were recruited via online advertisements and emails distributed among the inhabitants of Tsukuba City and its environs (a north-middle region in Japan) from November 2024 to February 2025. Emails were sent to those who had been registered in a database of potential participants. The advertisement informed participants that the study would take place at the National Institute of Advanced Industrial Science and Technology and assess psychological and behavioral dynamics in daily life. A total of 232 individuals were assessed for eligibility, of which 40 (mean age 45.40, SD 10.50 years) were enrolled. Recruitment occurred as part of an overarching project that encompassed smaller trials to test the feasibility and efficacy of different mHealth interventions [27]. For this trial, recruitment was combined with that for another trial [28], which required participation with a family or friend as a pair. Participants who were not eligible for that trial or could not find a time that worked for each of the pairs were invited to this trial. The inclusion criteria for this study were the same as those of the other trials in the project (see the paper by Takano et al [27] for the complete list): aged 18‐64 years, expecting no major life changes right before or during the study, not pregnant or lactating, in good health and receiving no medication (eg, tranquilizers or antihypertensive drugs), and not exercising regularly. We assumed that participants had good internet literacy, and necessary support was provided at the briefing session by a researcher. All participants were paid for their participation.

The sample size was determined pragmatically by considering the resources and capacity of the recruiting institute. With no suitable previous information on the expected size of the intervention effect when the study was conceptualized, we decided not to perform a priori power calculations. However, meta-analytic studies have estimated the effect size of text message interventions for PA as Cohen *d*=0.30‐0.40 (with or without message tailoring) [29]. A pilot trial reported an effect size of Cohen *d*=0.90, favoring personalized messages tailored to users’ PAs tracked by an app [26,30]. With the current sample size (n=40), an effect of Cohen *d*>0.91 can be detected reliably (with α=.05 and power=0.80). Although this size may be somewhat overly optimistic, rules of thumb suggest sampling n=30‐40 as the minimum for a pilot trial [31,32]; and if the main trial is considered together, the sample size for a pilot trial could be even smaller [33].

### Randomization and Blinding

Eligible participants were randomly allocated to either the intervention or control group in a 1:1 ratio. Block randomization was used (permutations within a batch of 4 participants) to balance the sample size between the 2 groups. A random allocation sequence was generated by a project member using R before recruitment. Experimenters (who conducted the recruitment and assessments) were not involved in this process. Single blinding was used; all participants received the same instructions (participants allocated to the control condition were also told that they might receive messages after responding to a prompt) and did not know what condition they were assigned to. Experimenters handed lab smartphones (programmed with the prompt schedules and messages to deliver) to participants, so that they knew the condition allocations. Yet, messages were automatically delivered, and researchers were not involved in the actual intervention.

### Interventions

Each participant received a smartphone and a Fitbit device at the beginning of the study and was asked to wear the Fitbit device on their nondominant wrist for 24 hours. The Fitbit device was linked to the phone via the Fitbit app, via which participants could monitor their step count and other physical data (eg, HR) at any time. Each evening, the participants were asked to self-report their daily step count and motivation for PA on an EMA app (m-Path; KU-Leuven [34]). The self-reported step count was meant to be a manipulation of self-monitoring and feedback (behavior change techniques taxonomy v1 [35]), controlling for the variability in attention (or preventing participants from paying no attention) to the PA and activity tracker.

Over the 4-week study duration, each participant was prompted by the EMA app to indicate (1) the current location (at home vs work or school vs other), (2) weather (sunny or cloudy vs rainy or snowy), and (3) social context (alone or with a family member, friend, colleague, or other). The EMA prompts were sent 3 times a day (at 8 AM, 12 PM, and 5 PM), and the participants could respond until the next prompt arrived. Only the EMI group received tailored messages upon completing each EMA questionnaire. This EMI began in the second week of the study period (the first week served as the baseline, with no EMI).

Messages were generated by combining sentences and phrases tagged with particular responses to the EMA questions. Specifically, when a participant indicated that the weather was nice, the EMI system used the stem sentence (Why not go out for some light exercise that…), followed by:

- … you can do when the weather is good? (at home)
- … you can do at work or school when the weather is good? (at the workplace or school)
- … can be done anywhere when the weather is good? (at other places)

Similarly, when a participant indicated that the weather was not good, the stem (Why not incorporate some light exercise that…) was combined with

- … you can do at home? (at home)
- … you can do at work or school? (at the workplace or school)
- … can be done anywhere? (other places)

Each message was followed by a mention of the social context as follows:

- Exercising with your family, friends, and colleagues is also effective in forming an exercise habit (being with a family, friend, or colleague).
- The following activities are as strenuous as or more strenuous than walking (being with other people or being alone).

For example, when a participant indicated that the weather was nice and they were alone at home, a message was generated as follows: “Why not go out for some light exercise that you can do when the weather is good? The following activities are strenuous or more strenuous than walking: (1) weeding the garden, cleaning around the entrance, (2) walking to the nearest park, and (3) hanging the futon (bed mattress) out in the sun, changing the bedsheet or linen.

The participants were presented with a set of activity recommendations that were deemed suitable for the indicated context (eg, outdoor activities recommended for good weather response; see Table 1), and the app asked them to indicate any activity that they would like to try then. If the participants were too busy to exercise at that moment, they could choose an option of “not now, maybe later” or “try another activity” for the sake of users’ autonomy [18]. The recommended activities were selected from an existing activity list [36] with known metabolic equivalents (METs), some of which were slightly modified to fit the EMI context (eg, “Walking for pleasure,” rephrased as “Walking around office or school, or shopping at a store that is a bit far”). We selected activities that (1) were not too hard, ranging from 3.0 to 7.0 hourly metabolic equivalents (METs-h/w), (2) could be performed either at home or at the office or school, and (3) were not too time-consuming and could be completed in 30 minutes or less. Also, all these activities could be performed alone or together with someone else; therefore, we did not select the type of activity depending on the social context specified in EMA (simply recommended co-working on the activity that fits the location and weather whenever a family or friend was present).

**Table 1.** Recommended activities for different locations and weather conditions.

Location	Weather	Activity^a^
Home	Sunny or cloudy	Weeding the garden, cleaning around the entrance (3.5 METs^b^). Walking to the nearest park (3.5 METs). Hanging the futon (bed mattress) out in the sun, changing the bedsheet or linen (3.3 METs).
Home	Rainy or snowy	Vacuuming (3.3 METs). Scrubbing the bathroom and bathtub (3.5 METs). General kitchen activity (eg, cooking, washing dishes, and cleaning up; 3.3 METs).
Work or school	Sunny or cloudy	Walking around the office or school, shopping at a store that is a bit far (3.5 METs). Workout in the nearest park or playground, with or without equipment (eg, park bench workout; 3.8 METs). Jogging (7 METs).
Work or school	Rainy or snowy	Walking fast in the office or school hallways (3.5‐4.3 METs). Going up and down the stairs (4 METs). Organizing or cleaning your desk area in the office (4.8 METs).
Other	—^c^	Using the stairs (instead of an elevator or escalator; 4 METs). Taking a short detour to the destination (3.5 METs). Walking faster than usual (3.5‐4.3 METs).

a The activity recommendations were not tailored to different social contexts, as each listed activity could be performed alone or with someone else.

b MET: metabolic equivalent.

c For the other locations, weather conditions were not used to tailor the activity recommendations. Instead, the recommendations were meant to be tips to increase PA when traveling from one place to another (eg, on the way home).

### Measures

We conducted 2 types of measurements, namely pre- and postintervention assessments, and the EMA. The primary outcome was self-reported PA before and after the intervention. Secondary outcomes included Fitbit measures (step count and HR) and motivations for PA. Self-reported PA was treated as the primary outcome because (1) the International Physical Activity Questionnaire—Short Form (IPAQ-SF) was a widely used measure that secures good comparability with other trials in the research field; and (2) we expected that the EMI would enhance motivation for PA, which would be more likely to be transferred to subjective than objective PA. Self-reported PA is not always consistent with the objective (eg, accelerometer-based) PA [37], and is known to be vulnerable to social desirability and other reporting biases [38]. Therefore, we included the Fitbit measures as the secondary outcomes to mitigate these limitations or even to cover the aspects that the subjective measure could not detect (eg, small changes that cannot be perceived).

### International Physical Activity Questionnaire—Short Form

Subjective PA levels were assessed as the primary outcome using IPAQ-SF [39,40]. This self-report scale has three activity categories: (1) walking, (2) moderate-intensity activity, and (3) vigorous-intensity activity (the inactivity category or sedentary time was not used in this study). The participants were asked to report the number of days and hours spent on each category in a typical week. The reported weekly hours of activities (days×hours) were aggregated across the 3 categories to represent total PA in the form of hourly metabolic equivalents (METs-h/w).

### Self-Determined Motivation Scale for Exercise

Motivation for PA was assessed as the secondary outcome pre- and postintervention using the revised version of the Self-Determined Motivation Scale for Exercise (SMSE) [41]. This scale has 22 items representing 6 types of motivations and regulatory styles for PA and exercises with the self-determination theory as the theoretical basis [42]. Some items were as follows: for intrinsic motivation, “exercising itself is fun;” for external regulation, “I exercise because other people will be pleased with me;” for introjected regulation, “I feel guilty if I do not exercise;” for identified regulation, “I think it is a good way to improve myself;” for integrated regulation, “it is essential to my identity and sense of self;” and for amotivation, “I do not know why I exercise.” Each item was rated on a 5-point scale (1=“Not at all true” and 5=“Very true”), and the sum of the scores was calculated for each motivation type. The Cronbach alphas for the current data were >0.64.

### Stage-of-Change Questionnaire

To assess the readiness for PA at baseline, we used the Stage-of-Change Questionnaire for PA [43,44]. This questionnaire asked participants to indicate the most applicable statement among the following 5 statements: “I currently do not exercise and do not intend to start exercising in the future” (precontemplation); “I currently do not exercise but I am thinking about starting to exercise in the next six months” (contemplation); “I currently exercise some but not regularly” (preparation); “I currently exercise regularly but have only begun doing so within the last six months” (action); and “I currently exercise regularly and have done so for longer than six months” (maintenance).

### Fitbit Measures

Daily step count and HR were assessed using a Fitbit device (Sense or Sense 2). Valid wear time was estimated by counting the frequency of valid HR values recorded (in minutes) over the 4-week study period. The HR was also analyzed as an outcome measure to evaluate the efficacy of this EMI, but this was not included in the preregistration protocol. We decided to analyze this as a secondary outcome because our EMI-recommended activities were physically more intensive than a sedentary daily routine but did not necessarily increase the step count (eg, kitchen activity). In processing the HR data, we first determined the target HR ranges with a threshold of 40% HRR or higher for each participant and then indexed the intensity of PA with the minutes of HR exceeding the threshold each day. The guidelines of the American College of Sports Medicine [45] classify 40%‐59% HRR as moderate intensity (METs 3‐5.9), which corresponds to the target PA intensity this EMI aimed to encourage. The threshold of 40% HRR was calculated using the following equation: (resting HR + [maximum HR–resting HR] × 0.4). The maximum HR was estimated using the Tanaka equation (208–participants’ age in years ×0.7) [46], in which the resting HR was the average defined by the Fitbit over the study period.

### Modified Situational Motivational Scale

The participants were prompted to respond to an EMA signal each evening (at 7 PM) in addition to the 3 EMA signals in the morning and afternoon. The evening EMA included items on current motivations for PA, each representing identified regulation (ie, “I’m feeling that exercising is important for me”), intrinsic motivation (ie, “I’m feeling that exercising is fun”), and external motivation (ie, “I’m feeling that I have to exercise”). These items were adapted from the Situational Motivational Scale [47] for physical exercise [48], with the wording modified for the EMA [27]. Each item was rated on a 7-point scale (1=“Not at all” and 7=“Very much”).

### Feasibility

At the postintervention assessment, participants in the EMI group were asked to rate the intervention as follows: the recommended activities and exercises were (1) satisfactory, (2) useful, (3) helpful, (4) suited to me, (5) conducted at an appropriate time, (6) practical, and (7) “I would like to continue using this intervention system” [49]. Each item was rated on a 7-point scale (1=“Not at all” and 7=“Very much”).

### Procedure

**Figure 1.**
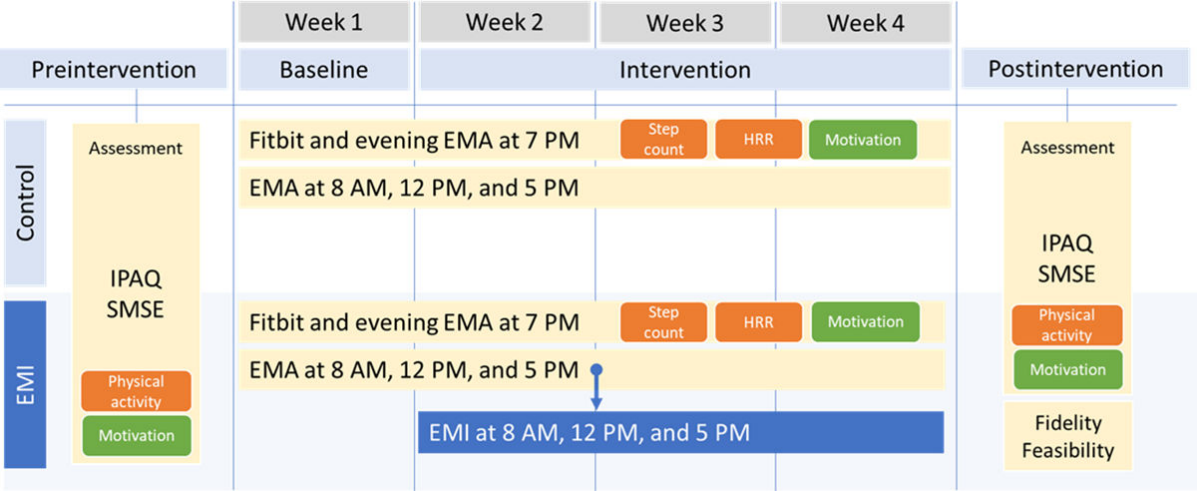
Study flowchart. EMI: ecological momentary intervention; EMA: ecological momentary assessment; HRR: heart rate reserve; IPAQ: International Physical Activity Questionnaire; SMSE: Self-Determined Motivation Scale for Exercise.

Figure 1 shows the study flowchart. Each eligible participant was invited to the laboratory, where they received the relevant study information and provided written informed consent. At this time, they completed the preintervention assessment, including the IPAQ-SF and SMSE-2. The day after this briefing session, the participants started the baseline week (week 1), during which they wore a Fitbit and responded to 3 daytime and 1 evening EMA prompts. The EMI started in week 2 and lasted until week 4; only the intervention group received a tailored message upon completing the EMA. The control group continued the EMA for this 3-week intervention period, but did not receive any messages from the app. The EMA prompts and EMI messages were sent automatically. Technical support was offered by a researcher whenever appropriate (eg, for app login and online questionnaires). After week 4, the participants completed the postintervention assessment for the outcomes (IPAQ-SF and SMSE-2) and feasibility (only the intervention group). The postintervention assessment took place 1.35 days (range 0‐9) after the completion of the intervention.

### Ethical Considerations

The protocol of the overarching study that included this study was approved by the Ethics Committee of the National Institute of Advanced Industrial Science and Technology (ID 2022‐1240). The protocol used in this study is available in the Open Science Framework [50]. We adhered to the CONSORT-EHEALTH (Consolidated Standards for Reporting Trials of Electronic and Mobile Health Applications and Online Telehealth) guidelines (see Checklist 1) [51] when reporting the study findings.

The participants received information about the procedure and aims of the study as well as their rights before completing the preintervention assessment. They were also explicitly informed that they had the right to withdraw from the study without any consequences. Subsequently, they provided written informed consent. Pseudo-anonymization was used to ensure data privacy and confidentiality. Participants’ personal identities were masked using unique participant IDs for the data collected through smartphones, activity trackers, and online questionnaire forms (for pre- and postintervention assessments). A reference list linking the participant IDs and personal information (eg, names and email) was created digitally. This list was only accessible by the first author and was destroyed at the end of data collection. Each participant was compensated at the end of the study (JPY 15,000‐20,000; US $100‐140).

### Statistical Analysis

The primary outcome (IPAQ total score) was analyzed using a multilevel model with the group (control vs EMI) as the between-person factor and time as the within-person factor (pre- vs postintervention). A random intercept and slope were assumed to account for individual differences in baseline levels and changes in PA. The same multilevel model was applied to the secondary outcomes assessed pre- and postintervention. Another set of multilevel models was estimated for the day-level outcomes assessed over the 3-week intervention period. Each model included groups as the between-person predictor, time (days since the beginning of the intervention) as the within-person predictor, and their interaction. In each model, the mean over the baseline week was controlled for, and a random slope was assumed to explain individual differences in the changes in outcomes. Restricted maximum likelihood estimation was used. We calculated Cohen *d* for group differences in (1) the postintervention scores and (2) means during the intervention period per week (ie, weeks 2‐4). All analyses were conducted using R (version 4.2.2; R Foundation for Statistical Computing), with the relevant packages of *lme4*, *lmerTest*, *emmeans*, and *fitbitr* [52-54].

Analyses were performed on an intention-to-treat basis; all randomized participants were included in each test. As no participants dropped out of the study, we had complete data for the variables assessed pre- and postintervention. For the EMA variables, we explored the variables that informed missingness before running the multilevel modeling analyses. Specifically, we calculated the compliance rates of the EMA responses for each participant, which were then tested for correlations with participant characteristics at baseline. As we found no significant correlations (see the compliance section), we did not use a covariate in each multilevel model.

## Results

### Recruitment, Demographics, and Baseline Characteristics

**Figure 2.**
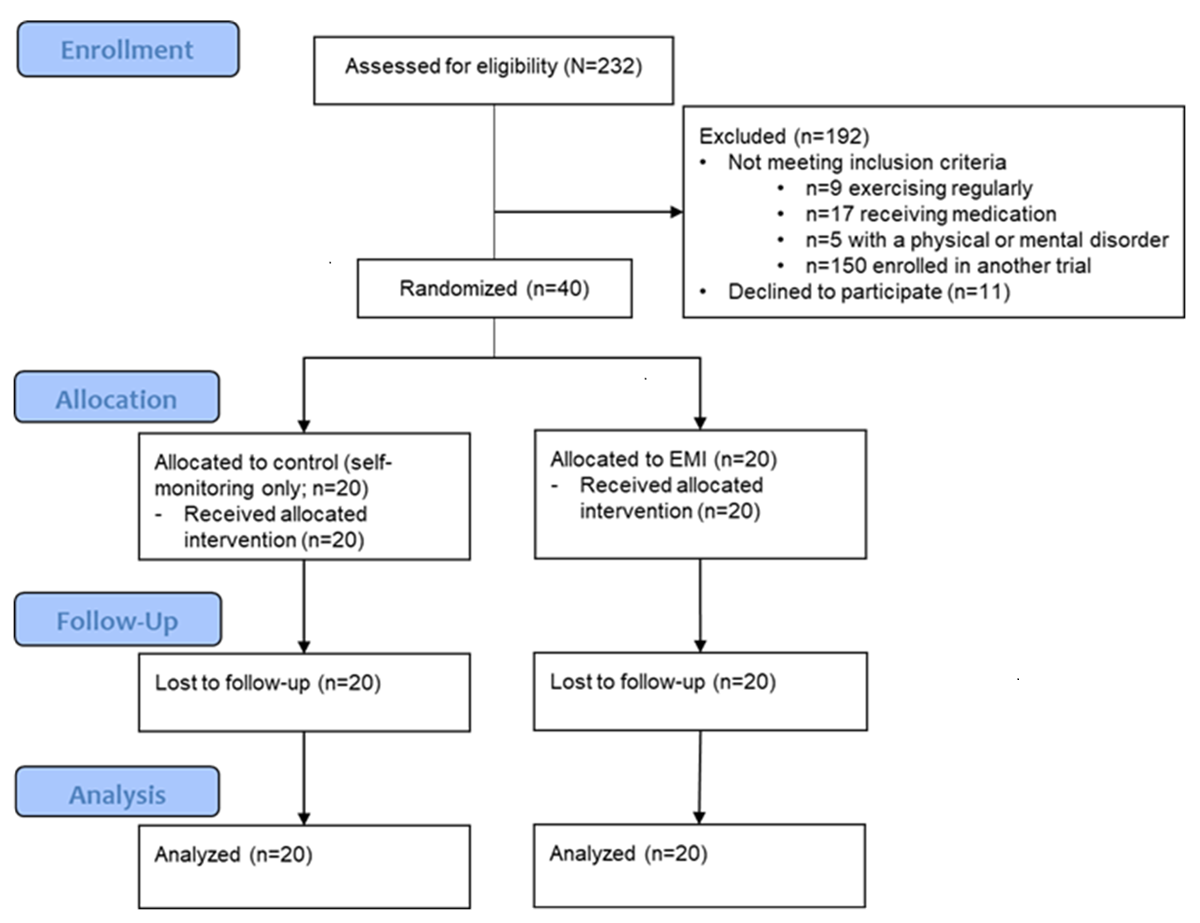
CONSORT (Consolidated Standards of Reporting Trials) flow diagram. EMI: ecological momentary intervention.

Figure 2 illustrates the recruitment process. The data collection was conducted between November 2024 and March 2025. All randomized participants (n=40) completed the postintervention assessment and were included in the analyses. No dropouts were observed, and no adverse events were reported (14 participants reported a light skin rash likely due to wearing a Fitbit). The baseline characteristics of the participants are summarized in Table 2. Most participants were middle-aged, had received higher education (graduated from a 2-year college, university, or higher), and had a job. Their BMI was in the normal range (18.5‐25.0 kg/m^2^), but they reported little to no habit of PA at baseline (ie, most were at a precontemplation or contemplation stage as per the Stage-of-Change Questionnaire). No significant differences were detected between the EMI and control groups in baseline characteristics, except for household income (higher in the EMI group), which was controlled for in subsequent analyses.

**Table 2.** Demographics and descriptive statistics at baseline.

Variables	Control (n=20)	EMI^a^ (n=20)	*t* test (*df*)	Chi-square (*df*)	*P* value
Age, mean (SD)	45.2 (8.08)	45.6 (12.69)	0.12 (38)	—^b^	.91
Women, n (%)	13 (65)	10 (50)	—	0.41 (1)	.52
BMI, mean (SD)	24.5 (5.06)	22.26 (3.69)	1.60 (38)	—	.12
Married, n (%)	13 (65)	12 (60)	—	0 (1)	≥.99
One or more children, n (%)	14 (70)	11 (55)	—	0.43 (1)	.51
Education level, n (%)			—	3.59 (2)	.17
Middle or high school	5 (25)	5 (25)			
College	12 (60)	7 (35)			
University and above	3 (15)	8 (40)			
Job, n (%)	19 (95)	17 (85)	—	0.28 (1)	.60
Household income, n (%)			—	12.75 (4)	.01
JPY <3 million (US $2000)	5 (25)	3 (15)			
JPY 3‐5 million (US $2000‐3333)	2 (10)	7 (35)			
JPY 5‐7 million (US $3333‐4667)	10 (50)	2 (10)			
JPY 7‐10 million (US $4667‐6667)	3 (15%)	4 (20)			
JPY 10 million and above (US $6667 and above)	0 (0)	4 (20)			
PA^c^ (METs-h/w)^d^, mean (SD)	21.63 (42.56)	9.45 (10.56)	1.24 (21.33)	—	.23
Stage of change, n (%)			—	4.40 (4)	.36
Precontemplation	6 (30)	5 (25)			
Contemplation	10 (50)	7 (35)			
Preparation	2 (10)	7 (35)			
Action	1 (5)	0 (0)			
Maintenance	1 (5)	1 (5)			
SMSE-2^e^, mean (SD)					
Intrinsic motivation (Cronbach α=0.75)	11.50 (3.56)	12.30 (3.34)	0.73 (38)	—	.47
Integrated regulation (Cronbach α=0.85)	9.70 (3.20)	10.50 (3.82)	0.72 (38)	—	.48
Identified regulation (Cronbach α=0.88)	14.25 (3.40)	14.20 (4.15)	0.04 (38)	—	.97
Introjected regulation (Cronbach α=0.86)	10.80 (4.18)	11.45 (3.97)	0.51 (38)	—	.62
External regulation (Cronbach α=0.64)	7.35 (2.54)	6.50 (2.44)	1.08 (38)	—	.29
Amotivation (Cronbach α=0.83)	6.90 (2.47)	6.40 (2.66)	0.62 (38)	—	.54
Baseline phase (week 1)					
Steps, mean (SD)	7639.84 (3767.33)	7071.18 (2843.90)	0.54 (38)	—	.59
Motivation, mean (SD)					
Intrinsic	3.79 (2.00)	4.35 (1.91)	0.91 (38)	—	.37
Identified	4.57 (1.96)	5.04 (1.82)	0.79 (38)	—	.44
External	4.43 (1.84)	5.24 (1.87)	1.37(38)	—	.18
HRR^f^ (min/d), mean (SD)	33.86 (29.52)	35.21 (23.13)	0.16 (38)	—	.87

a EMI: ecological momentary intervention.

b Not available.

c PA: physical activity.

d METs-h/w: hourly metabolic equivalents per week

e SMSE-2: Self-Determined Motivation Scale for Exercise.

f HRR: heart rate reserve.

### Compliance

The mean compliance rate of the EMA was 79.5% (SD 23.5%; 66.78 valid responses out of 3 prompts ×28 days), and that of the evening prompts was 87.2% (SD 18.81%). We detected valid HR signals with the Fitbit for 90.3% of the study duration (SD 10), indicating that the participants wore a Fitbit conscientiously during study participation. We found no statistically significant correlation between the compliance rates and baseline characteristics (see Table S1 in Multimedia Appendix 1).

### Efficacy of the Ecological Momentary Intervention

We performed multilevel modeling analyses with time and group to predict each outcome assessed at the pre- and postintervention time points. The estimated fixed effects can be found in Table S2 in Multimedia Appendix 1. The marginal mean changes and effect sizes (ie, standardized group differences in the change) are presented in Table 3. Although the EMI group showed slightly larger increases in PA than the control group (group difference=7.01), the statistical tests detected no significant group-time interaction effect on the primary (PA assessed by the IPAQ-SF; *P*=.44, Cohen *d*=0.21, 95% CI −0.35 to 0.78) or secondary (motivations assessed by the SMSE; from *P*=.11 to *P*=.91) outcomes.

**Table 3.** Marginal mean changes in physical activity and motivations between the control and ecological momentary intervention groups. Changes in pre- and postintervention assessments were calculated.

Outcome	Control (n=20), Estimate (95% CI)		EMI^a^ (n=20), Estimate (95% CI)		Group difference, Cohen *d* (95% CI)	
PA^b^ (METs-h/w)^c^	4.66 (−12.55 to 21.87)		11.67 (−5.54 to 28.88)		0.21 (−0.35 to 0.08)	
Motivation						
Intrinsic	0.15 (−1.17 to 1.47)		−1.00 (−2.32 to 0.32)		−0.28 (−0.63 to 0.01)	
Integrated	−0.30 (−2.00 to 1.40)		−0.40 (−2.10 to 1.30)		−0.03 (−0.49 to 0.04)	
Identified	0.10 (−1.48 to 1.68)		−0.45 (−2.03 to 1.13)		−0.12 (−0.49 to 0.03)	
Introjected	−0.20 (−2.17 to 1.77)		−0.75 (−2.72 to 1.22)		−0.12 (−0.59 to 0.04)	
External	−0.20 (−1.71 to 1.31)		−0.40 (−1.91 to 1.11)		−0.07 (−0.61 to 0.05)	
Amotivation	0.55 (−1.07 to 2.17)		0.10 (−1.52 to 1.72)		−0.15 (−0.71 to 0.041)	

a EMI: ecological momentary intervention.

b PA: physical activity.

c METs-h/w: hourly metabolic equivalents per week

Another set of multilevel modeling analyses was performed for the EMA outcomes, namely daily step count, motivation, and minutes of 40% HRR. In each model, the baseline (ie, week 1) levels were controlled, and the main and interaction effects of days and groups were tested. We found no significant main or interaction effects on any outcome (see Table S3 in Multimedia Appendix 1), except for a significant main effect of group on HRR (fixed effect=16.03; SE 6.44; *P*=.02; 95% CI 3.76 to 28.29; Cohen *d*=0.35, 95% CI 0.06 to 0.64). This effect implies that, compared with the control group, the EMI group showed larger increases in minutes of 40% HRR for the intervention (relative to the baseline) weeks (see Table 4). This group difference was prominent in the first 2 weeks (Cohen *d*=0.41, 95% CI 0.11 to 0.72; *P*=.01 and Cohen *d*=0.30, 95% CI 0.01 to 0.60; *P*=.04) but diminished in the last week of the intervention (Cohen *d*=0.20, 95% CI −0.17 to 0.56; *P*=.28).

We expected that tailored recommendations would enhance participants’ engagement with and motivation for PA. Therefore, we investigated how frequently each participant accepted the EMI-recommended activities and how acceptance frequency was associated with levels and motivations for PA. The EMI group reported that they would engage with (ie, accept) a recommended activity in response to 17.3 prompts (SD 17.02; 27.5% out of 3 prompts×21 days) over the intervention phase; they indicated “not now, maybe later” 28.25 times (SD 18.25; 44.8% out of 3 prompts×21 days) and “try another activity” 6.40 times (SD 7.29; 10.2% out of 3 prompts×21 days). The most frequently accepted activities were (1) weeding the garden and cleaning around the entrance (73/346, 21.1%), (2) using the stairs (61/346, 17.63%), and (3) hanging the futon (bed mattress) out in the sun and changing the bedsheet or linen (46/346, 13.3%; see Table S4 in Multimedia Appendix 1). The acceptance frequency showed a positive correlation with increases in the EMA-assessed identified regulation (*r*=.45, *P*=.047). Similarly, acceptance was positively correlated with increases in intrinsic motivation and integrated regulation, but this was not statistically significant (*r*=.41 and *r*=.36; *P*=.08 and *P*=.09, respectively; see Table S5 in Multimedia Appendix 1). No significant correlations were found with the changes in PA amount and intensity.

**Table 4.** Marginal means of the ecological momentary assessment outcomes for the control and intervention groups at each week of the intervention phase.

Outcome	Control (n=20), Estimate (95% CI)		EMI^a^ (n=20), Estimate (95% CI)		Group difference, Cohen *d* (95% CI)	
Steps/day						
Week 2	7327.68 (6576.67 to 8078.70)		7960.28 (7209.23 to 8711.34)		0.17 (−0.12 to 0.46)	
Week 3	7114.60 (6344.60 to 7884.61)		7666.41 (6897.38 to 8435.44)		0.15 (−0.15 to 0.45)	
Week 4	6901.53 (5730.75 to 8072.30)		7372.54 (6203.75 to 8541.33)		0.13 (−0.32 to 0.58)	
Motivation						
Intrinsic						
Week 2	4.28 (4.03 to 4.53)		4.14 (3.89 to 4.39)		−0.16 (−0.60 to 0.27)	
Week 3	4.21 (3.93 to 4.49)		4.11 (3.83 to 4.39)		−0.11 (−0.59 to 0.37)	
Week 4	4.14 (3.79 to 4.49)		4.08 (3.74 to 4.43)		−0.06 (−0.65 to 0.53)	
Identified						
Week 2	4.75 (4.49 to 5.02)		4.96 (4.70 to 5.22)		0.24 (−0.20 to 0.68)	
Week 3	4.74 (4.44 to 5.03)		4.97 (4.67 to 5.27)		0.27 (−0.22 to 0.77)	
Week 4	4.72 (4.36 to 5.08)		4.98 (4.63 to 5.33)		0.30 (−0.29 to 0.89)	
External						
Week 2	4.97 (4.73 to 5.21)		4.92 (4.68 to 5.16)		−0.05 (−0.45 to 0.35)	
Week 3	4.98 (4.73 to 5.23)		4.88 (4.63 to 5.13)		−0.12 (−0.54 to 0.29)	
Week 4	5.00 (4.67 to 5.33)		4.84 (4.51 to 5.16)		−0.19 (−0.73 to 0.35)	
HRR^b^						
Week 2	22.95 (13.16 to 32.73)		41.70 (31.96 to 51.45)		0.41 (0.11 to 0.72)	
Week 3	23.78 (14.34 to 33.23)		37.68 (28.38 to 46.98)		0.30 (0.01 to 0.60)	
Week 4	24.62 (12.73 to 36.51)		33.66 (22.07 to 45.25)		0.20 (−0.17 to 0.56)	

a EMI: ecological momentary intervention.

b HRR: heart rate reserve; minutes of 40% HRR per day.

### Feasibility

For feasibility, each item was rated on a 7-point scale, and we interpreted scores of 4 or higher as an indication that participants agreed on that point. Approximately half of the participants in the EMI group reported that the intervention was satisfactory (10/20, 50%), useful (9/20, 45%), helpful (9/20, 45%), suitable (8/20, 40%), and practical (10/20, 50%; see Table 5). Only a quarter (5/20, 25%) rated the prompt timing as appropriate, implying that typical EMA prompting (here, 3 times a day with a fixed schedule) may not be suitable for the user experience. Despite the timing issue, more than half (11/20, 55%) of the participants were willing to continue the intervention.

**Table 5.** Feasibility rated by participants in the ecological momentary intervention group (N=20).

Item^a^	Values, mean (SD)	Endorsement, n (%)
Satisfactory	3.10 (1.21)	10 (50)
Useful	3.30 (1.08)	9 (45)
Helpful	3.10 (1.21)	9 (45)
Suitable	3.15 (1.04)	8 (40)
Timing	2.85 (1.04)	5 (25)
Practical	3.30 (1.03)	10 (50)
Willingness to continue	3.35 (1.46)	11 (55)

a Each item was rated on a 7-point scale (1=“Not at all” and 7=“Very much”). Scores of 4 or higher were regarded as endorsements for each item.

## Discussion

### Principal Findings

This pilot randomized controlled trial investigated the efficacy and feasibility of the EMI in improving PA, with messages and recommendations tailored to participants’ EMA responses. Overall, EMI had null effects on self-reported PA, motivation for PA, or step count as assessed by an activity tracker. However, the minutes of 40% HRR (moderate- or high-intensity PA) showed a larger increase in the EMI group than in the control group. Regarding feasibility, approximately half of the participants found the intervention useful and satisfactory and expressed a willingness to continue. However, the prompt timing was perceived as inappropriate, leaving room for improvement in the design and implementation.

### Efficacy

Contrary to our hypothesis, no significant improvement was observed in the self-reported PA or step count. These null findings are in line with the results of previous EMI trials [16-18], which failed to establish a transfer effect on PA. This result suggests that fully automated, low-intensity EMI may not be strong enough to induce a significant change in PA. Our EMI was delivered for 3 weeks, which was comparable to the average duration of EMIs in the literature; a systematic review documented that the duration of EMI trials ranged from 2 to 15 weeks, with an average of 4 weeks [1]. However, a JITAI study [55] showed that participants became bored even with contextually tailored messages (eg, “Is there anything better than weekend afternoons? You could enjoy the fresh air by taking a walk around your neighborhood!”) so that the effect of messages declined as the intervention progressed. That means EMI (or messaging in general) might have low intensity, needing some add-ons that maintain and facilitate active user engagement, like team competition or other forms of gamification [22]. Some more reflections in light of the principles proposed by Van Dantzig et al [20] can help in the future development of an EMI. First, we sent out 3 EMA prompts across the day, which was also comparable to the average sending frequency reported in the literature (average of 4 to 5 times [1]). However, this prompting might be too sparse to spot a moment of opportunity for individuals to engage in a behavior. Similar to other EMI trials (eg, using accelerometers to monitor and detect sedentary behavior [19,56]), integrating accelerometers and other wearable sensors into the EMI decision system to determine what messages to send and when might have improved acceptability and efficacy. Another possible technical improvement would be to link an EMI app to an external database through an application performance interface to fetch relevant momentary information (eg, weather and location) when needed, without relying on a user’s self-report [21,26]. Second, the recommended activities were carefully selected when designing the intervention, and we were convinced that each activity was actionable in each target context. However, we did not tailor the recommendations to individual preferences; the same decision rules and activity lists were used across participants. To improve acceptability, a user-defined activity list that aligns with user preferences [7] or frequent behaviors [26] may be more appropriate.

Notably, our EMI failed to establish an effect on motivation for PA, unlike earlier EMI trials [6,7,16,17]. We assumed that short, concise text messages would be sufficient to motivate participants as long as they were context-aware [16]. However, our findings may suggest that the prompting messages should contain explicit motivational and congratulatory words (delivering congratulatory and supportive messages when users engage in healthy behavior [6] and delivering personalized feedback and encouragement based on individuals’ predetermined goals [7]) to exert a positive psychological influence on message recipients. The causal relationship remains unclear (ie, whether EMI-induced motivation leads to an improvement in PA [16,17]), but motivation plays an important role in aspects other than efficacy, such as sustained user engagement and retention [57]. Developers of EMI should consider embedding a motivational source in their intervention if it is not personalized or tailored. In addition to motivation, the COM-B model highlights the roles of capability and opportunity as active components of an effective intervention for behavior change [13]. Our EMI was designed to help find an opportunity to perform PA in daily life, and the recommended activities did not require high levels of capability. However, half of the participants rated prompt timing as inappropriate, implying that the opportunity component did not work as intended. Inappropriate prompt timing may affect motivation to maintain the engagement—that means, through the lens of the COM-B model, the motivation and opportunity components would be the key challenges for the EMI.

We found that the EMI group showed a significantly larger increase in minutes of 40% HRR than the control group. Although the HRR analysis was exploratory (not preregistered), this significant increase may imply that the EMI group successfully integrated the recommended activities into their daily routine, which were small but slightly physically intensive behavioral changes. It should be noted that the EMI had no significant effect on step count or motivation outcomes, meaning that HRR changed independently of the amount of PA and motivation for that. A meta-analysis showed that autonomous motivation is not associated with the amount of PA (and likely the intensity as well) when assessed as an outcome in intervention studies [58]. In the context of physical education, HR-based intensity was found to have even a negative association with autonomous motivation [59]. These findings may suggest that individuals do not have to be intrinsically motivated to perform PA in an intervention. Our EMI recommended low-intensity PA that could be part of house choring (eg, cleaning, kitchen activity, and weeding the garden), which were necessary for living but did not have to be liked. Also, these activities did not require massive body movement, directly increasing step count. Although this narrative includes some speculation, it is possible that engaging in the recommended activities led to changes in HRR, while these changes were not reflected in motivation or step count.

### Feasibility

Compliance was excellent, and no dropouts were observed. However, the EMI was rated low for satisfaction and other feasibility items. Around half of the EMI participants found that the intervention was satisfactory, useful, helpful, suitable, and practical and showed a willingness to continue the intervention. For one-third of the EMA prompts, the participants indicated that they would engage in a recommended activity; for the other prompts, they typically indicated “not now, maybe later.” Indeed, prompt timing was perceived as suboptimal (no more than 25% of the participants rated the timing as appropriate). A pilot EMI trial [7] reported that participants found most (>80%) EMI messages enjoyable and motivating. Other trials have also documented that EMI messages and reminders are liked and accepted by participants [15,16]; however, several studies have noted that just-in-time messages might be perceived as too early or too late in a day [60] and that the activity recommendations might not always reflect participants’ preferences [26]. As we found a preliminary association between the frequency of accepting activity recommendations and the motivation outcome of integrated regulation (*r*=0.45), it is crucial to improve the acceptability of each message and recommendation. In this sense, using a more positive and encouraging tone in each message and tailoring recommendations per users’ preferences might be helpful. In addition, a machine learning approach may be able to resolve the optimization issues for improving acceptability. A recent study [61] trained a machine learning model on EMA data to predict momentary engagements in PA using situational and contextual information. Such a model’s prediction would allow for tailoring the content and timing of intervention prompts (eg, delivering motivational messages with specific suggestions when the highest message acceptability is predicted).

### Limitations

These results should be interpreted in light of the several limitations of this study. First, the sample size might not have been sufficiently large to detect several important effects, particularly for the correlation analyses within the EMI group. Second, the generalizability of the findings might have been limited by the homogeneity of the sample. We did not find any effects unique to the Japanese population, but some aspects of the EMI, such as the selection of recommended activities, might require appropriate adaptations for users with a specific cultural background and lifestyle. Third, the participants were paid volunteers, which might explain their good compliance and zero dropout rate. In a naturalistic setting, low retention rates are one of the most important challenges for mobile devices, especially for commercial health care apps [62,63]. A motivational source for incentivizing users is crucial when introducing the EMI to the market [64]. Fourth, we relied on self-reported EMA to assess the 3 tailoring variables. The self-reporting could be replaced with passive sensing (eg, GPS and audio recording), which would allow for just-in-time adaptive triggers without any cost for users to respond to EMA signals. For this study, we could not, however, clear data protection issues concerning passive sensing, especially for handling personal information (eg, GPS signals and voices). We see technical and ethical challenges in implementing a ubiquitous sensing system, but this will also resolve most of the timing issues in EMI. We used fixed-time prompting to improve the response rate of participants, but there would be no need to fix or define any prompt timing if real-time sensing becomes available.

### Conclusion

Regardless of these limitations, we believe that this pilot trial serves as an EMI exemplar. It highlights several important challenges in designing a successful EMI program, such as prompt timing and other technical or technological mechanisms to effectively detect a moment of opportunity, the message tone to be used (positive and motivational), and activity recommendations tailored to momentary contextual information and individual preferences. As noted above, machine learning may resolve part of the optimization issues for the timing and content of messaging. Another important direction is the use of passive sensing, which would require less or even no self-reporting to detect the moment of opportunity for intervention. Self-reported EMA may feel, even if carefully personalized, somewhat distracting and invasive due to the high frequency of prompting. These 2 approaches could be used in combination and have a large potential to improve the acceptability of intervention messages. This study also had an implication for the trial design, particularly for outcome selection. The EMI often targets daily routine behaviors (eg, sedentary time) and aims to replace them with slightly more active alternatives (eg, taking a break or standing). Such small behavioral changes may not be reflected in accelerometer-based outcomes but could be captured by HR. Although HRR has not been widely used in mobile assessments, future EMI trials may benefit from it, increasing the chances of identifying an undetected effect with step count and other PA amount measures.

## Supplementary material

10.2196/79360 Multimedia Appendix 1 Supplementary tables for each analysis.

10.2196/79360 Checklist 1 CONSORT checklist.

## Abbreviations

COM-B: capability, opportunity, motivation, and behavior
CONSORT-EHEALTH: Consolidated Standards for Reporting Trials of Electronic and Mobile Health Applications and Online Telehealth
EMA: ecological momentary assessment
EMI: ecological momentary intervention
HR: heart rate
HRR: heart rate reserve
JITAI: just-in-time adaptive intervention
MET: metabolic equivalent
PA: physical activity
SMSE: Self-Determined Motivation Scale for Exercise
