# Supplementary material for: Mobile-Based Ecological Momentary Intervention for Improving Physical Activity in Adults Without Regular Physical Activity: Pilot Randomized Controlled Trial
Source: JMIR Form Res. 2025 Dec 4;9:e79360. doi: 10.2196/79360 (PMC12677381; doi:10.2196/79360)
Supplement: Multimedia Appendix 1 [file formative-v9-e79360-s001.docx]

**Table S1.** Correlations (Spearman) between EMA compliance rates and baseline characteristics.

| Variable | Valid wear time  (Fitbit) | Compliance EMA | Compliance EMA  (evening signal) |
| --- | --- | --- | --- |
| Valid wear time (Fitbit) | 1.000 | 0.306 | 0.443 |
| Compliance EMA | 0.306 | 1.000 | 0.650 |
| Compliance EMA (evening) | 0.443 | 0.650 | 1.000 |
| Age | -0.006 | 0.196 | 0.140 |
| Gender | 0.234 | 0.101 | -0.039 |
| BMI | -0.262 | -0.238 | -0.117 |
| Marital status | -0.119 | 0.175 | 0.124 |
| One or more children | -0.177 | 0.202 | 0.164 |
| Education level | 0.301 | 0.145 | 0.234 |
| Job | -0.108 | 0.094 | 0.106 |
| Household income | 0.004 | 0.169 | 0.225 |
| PA (METs-h/w) | 0.275 | 0.216 | 0.107 |
| Stage of Change | -0.009 | 0.124 | -0.095 |
| Intrinsic motivation | -0.051 | 0.016 | -0.154 |
| Integrated regulation | 0.110 | 0.073 | -0.085 |
| Identified regulation | 0.149 | 0.227 | -0.017 |
| Introjected regulation | 0.308 | 0.259 | 0.239 |
| External regulation | -0.040 | -0.040 | -0.018 |
| Amotivation | -0.057 | -0.259 | -0.003 |

Note. EMA = Ecological momentary assessment; PA = Physical activity (Metabolic equivalents, hours/week)

**Table S2.** Estimates of multilevel modeling for the outcomes assessed at the pre- and post-intervention timing.

| Predictor | Estimate | SE | *t* | *P* | 95%CI |
| --- | --- | --- | --- | --- | --- |
| DV: PA |  |  |  |  |  |
| Intercept | 14.524 | 11.614 | 1.251 | 0.218 | [-8.032, 37.357] |
| Household income | 2.786 | 3.662 | 0.761 | 0.452 | [-4.422, 9.995] |
| Group | -13.295 | 9.875 | -1.346 | 0.186 | [-32.351, 5.888] |
| Time | 4.662 | 6.406 | 0.728 | 0.471 | [-7.875, 17.199] |
| Group × Time | 7.008 | 9.059 | 0.774 | 0.444 | [-10.722, 24.738] |
| DV: Intrinsic motivation |  |  |  |  |  |
| Intercept | 11.683 | 1.393 | 8.389 | < .001 | [8.993, 14.372] |
| Household income | -0.072 | 0.452 | -0.158 | 0.875 | [-0.944, 0.801] |
| Group | 0.829 | 1.121 | 0.739 | 0.464 | [-1.336, 2.994] |
| Time | 0.150 | 0.49 | 0.306 | 0.761 | [-0.809, 1.109] |
| Group × Time | -1.150 | 0.693 | -1.659 | 0.105 | [-2.506, 0.206] |
| DV: Integrated regulation |  |  |  |  |  |
| Intercept | 9.590 | 1.385 | 6.922 | < .001 | [6.848, 12.304] |
| Household income | 0.043 | 0.444 | 0.097 | 0.923 | [-0.839, 0.926] |
| Group | 0.783 | 1.142 | 0.685 | 0.497 | [-1.434, 2.986] |
| Time | -0.300 | 0.633 | -0.474 | 0.639 | [-1.540, 0.940] |
| Group × Time | -0.100 | 0.896 | -0.112 | 0.912 | [-1.853, 1.653] |
| DV: Identified regulation |  |  |  |  |  |
| Intercept | 13.032 | 1.512 | 8.620 | < .001 | [10.111, 15.953] |
| Household income | 0.478 | 0.491 | 0.974 | 0.336 | [-0.471, 1.426] |
| Group | -0.241 | 1.217 | -0.198 | 0.844 | [-2.591, 2.109] |
| Time | 0.100 | 0.588 | 0.170 | 0.866 | [-1.051, 1.251] |
| Group × Time | -0.550 | 0.832 | -0.661 | 0.512 | [-2.177, 1.077] |
| DV: Introjected regulation |  |  |  |  |  |
| Intercept | 9.512 | 1.581 | 6.018 | < .001 | [6.456, 12.562] |
| Household income | 0.505 | 0.506 | 0.998 | 0.325 | [-0.473, 1.483] |
| Group | 0.448 | 1.306 | 0.343 | 0.734 | [-2.079, 2.972] |
| Time | -0.200 | 0.734 | -0.273 | 0.787 | [-1.636, 1.236] |
| Group × Time | -0.550 | 1.037 | -0.530 | 0.599 | [-2.580, 1.480] |
| DV: External regulation |  |  |  |  |  |
| Intercept | 5.463 | 0.931 | 5.867 | < .001 | [3.619, 7.288] |
| **Household income** | **0.740** | **0.298** | **2.481** | **0.018** | **[0.147, 1.333]** |
| Group | -1.146 | 0.769 | -1.49 | 0.145 | [-2.639, 0.338] |
| Time | -0.200 | 0.562 | -0.356 | 0.724 | [-1.300, 0.900] |
| Group × Time | -0.200 | 0.795 | -0.252 | 0.803 | [-1.755, 1.355] |
| DV: Amotivation |  |  |  |  |  |
| Intercept | 6.956 | 0.997 | 6.976 | < .001 | [5.026, 8.879] |
| Household income | -0.022 | 0.318 | -0.069 | 0.946 | [-0.636, 0.593] |
| Group | -0.491 | 0.831 | -0.591 | 0.558 | [-2.100, 1.115] |
| Time | 0.550 | 0.601 | 0.915 | 0.366 | [-0.627, 1.727] |
| Group × Time | -0.450 | 0.850 | -0.529 | 0.600 | [-2.114, 1.214] |

**Table S3.** Estimates of multilevel modeling for the outcomes assessed during the intervention weeks

| Predictor | Estimate | SE | *t* | *P* | 95%CI |
| --- | --- | --- | --- | --- | --- |
| DV: Steps |  |  |  |  |  |
| Intercept | 1091.217 | 817.944 | 1.334 | 0.191 | [-514.531, 2700.798] |
| Household income | -24.677 | 196.408 | -0.126 | 0.901 | [-405.418, 356.091] |
| **Baseline** | **0.840** | **0.074** | **11.371** | **< .001** | **[0.697, 0.984]** |
| Group | 587.349 | 490.862 | 1.197 | 0.239 | [-347.802, 1522.959] |
| Time | -184.775 | 262.094 | -0.705 | 0.485 | [-697.799, 328.082] |
| Group × Time | -70.062 | 370.441 | -0.189 | 0.851 | [-794.98, 655.024] |
| DV: Intrinsic motivation |  |  |  |  |  |
| Intercept | 0.672 | 0.277 | 2.43 | 0.02 | [0.139, 1.202] |
| Household income | -0.071 | 0.071 | -0.998 | 0.325 | [-0.207, 0.066] |
| **Baseline** | **0.927** | **0.046** | **20.314** | **< .001** | **[0.84, 1.014]** |
| Group | -0.113 | 0.185 | -0.613 | 0.544 | [-0.467, 0.239] |
| Time | -0.062 | 0.05 | -1.255 | 0.217 | [-0.158, 0.036] |
| Group × Time | 0.036 | 0.07 | 0.521 | 0.605 | [-0.101, 0.172] |
| DV: Identified regulation |  |  |  |  |  |
| Intercept | 0.343 | 0.294 | 1.167 | 0.25 | [-0.217, 0.903] |
| Household income | -0.050 | 0.075 | -0.664 | 0.511 | [-0.195, 0.096] |
| **Baseline** | **0.946** | **0.049** | **19.125** | **< .001** | **[0.851, 1.042]** |
| Group | 0.222 | 0.196 | 1.131 | 0.265 | [-0.152, 0.597] |
| Time | -0.015 | 0.041 | -0.359 | 0.721 | [-0.096, 0.066] |
| Group × Time | 0.023 | 0.058 | 0.403 | 0.689 | [-0.09, 0.136] |
| DV: External regulation |  |  |  |  |  |
| Intercept | 0.512 | 0.257 | 1.996 | 0.054 | [0.025, 0.975] |
| Household income | -0.053 | 0.067 | -0.796 | 0.431 | [-0.15, 4.975] |
| **Baseline** | **0.953** | **0.045** | **20.962** | **< .001** | **[0.865, 0.975]** |
| Group | -0.079 | 0.166 | -0.475 | 0.638 | [-0.35, 6.975] |
| Time | 0.016 | 0.060 | 0.261 | 0.796 | [-0.15, 7.975] |
| Group × Time | -0.053 | 0.084 | -0.629 | 0.534 | [-0.25, 5.975] |
| DV: HRR |  |  |  |  |  |
| Intercept | 3.598 | 8.965 | 0.401 | 0.691 | [-13.754, 20.917] |
| Household income | 1.513 | 2.576 | 0.587 | 0.561 | [-3.45, 6.475] |
| **Baseline** | **0.464** | **0.124** | **3.757** | **0.001** | **[0.227, 0.702]** |
| Group | **16.033** | **6.436** | **2.491** | **0.017** | **[3.758, 28.292]** |
| Time | 0.725 | 2.313 | 0.313 | 0.756 | [-3.797, 5.246] |
| Group × Time | -4.213 | 3.242 | -1.299 | 0.202 | [-10.551, 2.128] |

**Table S4.** Frequency of acceptance of recommended activities.

| Activity | Frequency, n (%) |
| --- | --- |
| Weeding garden, cleaning around entrance | 73 (21.10) |
| Using stairs (instead of an elevator or escalator) | 61 (17.63) |
| Hanging futon (bed mattress) out in the sun, changing bed sheet/linen | 46 (13.30) |
| Walking to the nearest park | 43 (12.43) |
| Walking around office/school, shopping at a store a bit long way away | 40 (11.56) |
| Walking faster than usual | 35 (10.12) |
| Taking a short detour to the destination | 19 (5.49) |
| Kitchen activity, general | 10 (2.89) |
| Vacuuming | 6 (1.73) |
| Scrubbing bathroom and bathtub | 4 (1.16) |
| Workout in the nearest park or playground, with/out equipment | 3 (0.87) |
| Walking fast in office/school hallways | 2 (0.58) |
| Going up and down stairs | 2 (0.58) |
| Jogging | 1 (0.29) |
| Organizing/cleaning your desk area in office | 1 (0.29) |

**Table S5.** Correlations between the changes in each outcome and the frequency of accepted recommendations (N=20).

| Outcome | r | p |
| --- | --- | --- |
| Pre- to post-intervention |  |  |
| PA | -0.171 | 0.471 |
| Motivation |  |  |
| Intrinsic motivation | 0.407 | 0.075 |
| Integrated regulation | 0.385 | 0.094 |
| Identified regulation | 0.257 | 0.273 |
| Introjected regulation | -0.031 | 0.897 |
| External regulation | 0.361 | 0.118 |
| Amotivation | -0.268 | 0.253 |
| Baseline to intervention weeks |  |  |
| Step count | 0.101 | 0.671 |
| Motivation |  |  |
| Intrinsic motivation | 0.118 | 0.620 |
| Identified regulation | 0.450 | 0.047 |
| External regulation | 0.004 | 0.988 |
| HRR | -0.043 | 0.856 |

Note. Changes were defined as: [post-intervention score] minus [pre-intervention score]; or [mean of the intervention week] minus [mean of the baseline week].
